# The effect of mannitol on postoperative renal function in patients undergoing coronary artery bypass surgery: A double-blinded randomized controlled trial

**DOI:** 10.34172/jcvtr.32992

**Published:** 2024-09-20

**Authors:** Masumeh Hemmati Maslakpak, Eisa Bilejani, Sohrab Negargar, Ahmadali Khalili, Vahid Alinejad, Amir Faravan

**Affiliations:** ^1^Maternal and Childhood Obesity Research Center, Nursing and Midwifery School, Urmia University of Medical, Urmia, Iran; ^2^Department of Anesthesia, Faculty of Medicine, Tabriz University of Medical Sciences, Tabriz, Iran; ^3^Cardiovascular Research Center, Tabriz University of Medical Sciences, Tabriz, Iran; ^4^Department of Biostatistics, Urmia University of Medical Sciences, Urmia, Iran; ^5^Student Research Committee, Tabriz University of Medical Sciences, Tabriz, Iran

**Keywords:** Cardiopulmonary bypass, Mannitol, Coronary artery bypass, Ringer’s lactate

## Abstract

**Introduction:**

Mannitol, an osmotic diuretic solution, is commonly utilized in priming cardiopulmonary bypass (CPB) and can impact kidney function. This study was conducted to investigate the impact of mannitol use during CPB on kidney function in patients undergoing coronary artery bypass surgery.

**Methods:**

This randomized, double-blind clinical trial studied 90 patients undergoing coronary artery bypass surgery. In the control group (n=45), the prime solution included Ringer’s lactate, and in the intervention group (n=45), the prime solution had 200 ml of mannitol 20% and Ringer’s lactate. A *P*-value<0.05 was considered significant. The primary endpoint of this study is renal function.

**Results:**

Demographic characteristics and risk factors were not significantly different between the two groups (*P*>0.05). Additionally, there was no statistically significant difference between two groups in terms of CPB time, aortic cross-clamp time, length of time connected to mechanical ventilation, 30-day mortality, ICU, and hospital stay time (*P*>0.05). Furthermore, no statistically significant difference was observed between the two groups in serum creatinine levels (*P*=0.53) or BUN levels (*P*=0.13). The study also found no statistically significant difference in the diuresis rate between the two groups (*P*=0.10).

**Conclusion:**

The present study has shown that adding mannitol to the prime has no effect on kidney function, length of time connected to mechanical ventilation, length of stay in the ICU, or 30-day mortality. Therefore, it suggests that mannitol cannot be used as a preventative strategy for acute kidney injury after cardiac surgery.

## Introduction

 Most cardiac surgeries require cardiopulmonary bypass (CPB). The CPB system provides both circulatory and respiratory supports. The physiology of CPB is not exactly same as native heart and lung function and can result in inflammatory, hematologic, immunologic, and microembolic injuries in patients.^1^ An important step in preparing and setting of CPB circuit is its priming, which can affects postoperative CPB complications. ^2^ The fluid solution used for priming is a mixture of crystalloids and colloids.^3^ Many additives can be added to this solution such as mannitol, sodium bicarbonate, albumin and blood products in propose of attenuating distracting effects of extracorporeal circulation. Presently, mannitol is the most commonly used additive and due to its osmodiuretic effects, it provides a higher blood oncotic pressure.^4-6^ This high blood oncotic pressure reduces fluid leak to third space and helps to maintain fluid balance during bypass procedure and postoperative diuresis.^7^

 There are many studies those investigated the effects of adding mannitol to priming solution on the postoperative renal function; however the results were contradictory and it was suggested that more researches are needed to confirm its probably beneficial effects.^3,8-10^ Hamiko et al reported that priming with mannitol associated with reduced incidence of postoperative delirium, decreased mechanical ventilation time, ICU and hospital stay, and lower treatment costs.^11^ Additionally, mannitol can induce renal vasodilation and redistribution of systemic blood flow to the kidneys.^12^ The increased renal blood flow may improve oxygenation and glomerular filtration rate and prevent acute kidney injury and improve kidney function.^13,14^ In a clinical trial, Ljunggren et al reported that adding mannitol to priming solution did not affect hemoglobin level, blood acidity, bicarbonate, potassium and chloride levels. Despite increased preoperative diuresis mannitol did not have any renal protection effect.^15^ Whitta et al in a study on patient undergoing liver transplantation, concluded that intraoperative mannitol does not have any renal protection property.^16^ Haydock et al demonstrated that removing mannitol from priming solution did not have any detrimental effect on outcome in patients having primary isolated CABG surgery.^17^ Khademi et al in patients undergoing elective CABG with CPB reported that there is not any correlation between diuresis during CPB and change in postoperative renal function.^18^ Additionally, hyperosmolar priming solutions may cause a notable and rapid rise in plasma osmolality. This increase can lead to organ dysfunction, including osmotic demyelination syndrome.^19^

 Considering these contradictory results due to mannitol in CPB priming, we conducted this present study to investigate the effect of mannitol in CPB priming on renal function in patients undergoing elective coronary artery bypass graft surgery.

## Materials and Methods

###  Study design

 This study is a single-center, double-blind, and randomized controlled trial approved by the ethics committee of Urmia University of Medical Sciences (IR.UMSU.REC.1401.319). In this study, from December 2022 to May 2023, patients aged 18-70 who underwent coronary artery bypass surgery at Shahid Madani Heart Center were studied. The inclusion criteria were a Left ventricular ejection fraction above 30% in echocardiography before surgery and normal renal function. Exclusion criteria included acute surgery, a history of cardiac surgery, documented allergic reactions, documented psychiatric or dementia issues, a body mass index greater than 40 kg/m2, the need for deep hypothermia, and severe vision or hearing problems.

 We thoroughly explained the research objectives and potential benefits and harms to the patients. If they chose to participate in the research, they completed and signed the written informed consent form. Each patient had the right to refuse to cooperate and withdraw from the study at any point up until anesthesia induction. Furthermore, no additional costs were incurred by the patient during this study. The primary endpoint of this study is renal function.

 The sample size was calculated using data from Ljunggren et al’s study^15^, with a power of 80% and alpha of 0.05. Accounting for a 25% attrition rate, 45 samples were obtained for each group using the following formula:

 n = 2δ^2^(z_1-α/2_ + z_1- β_)^2^/(µ_1_-µ_2_)^2^

 The patient sampling process was meticulously conducted, with patients being randomly assigned to either the control or intervention groups. A total of ninety sealed, numbered opaque envelopes, each containing a unique code, were prepared for each group (n = 45). Patients were then randomly selected an envelope just before entering the operating room, and the corresponding code was assigned to them. Patients were allocated to two control and intervention groups at a ratio of 1:1. In case if a patient needed to be removed from the study, the same code was placed back into the pool of envelopes, ensuring the maintenance of the desired sample size for analysis. Prior to the surgery, comprehensive basic information such as renal function tests and medication history was diligently recorded. The patient’s medical records were thoroughly reviewed to identify any history of mental illness. This care was meticulously standardized, ensuring no variance between the two groups. The process of randomization and allocation concealment was carried out by an individual who had no other involvement in conducting the study or analyzing the data.

###  Perfusion technique

 CPB was performed using an artificial heart and lung machine, specifically the Stöckert S5® model roller pumps and INSPIRE® 8f membrane oxygenator. When the surgeon ordered the preparation of the extracorporeal circulation circuit, a perfusionist colleague designed a intervention drug (200 ml serum). The serum was then covered with opaque adhesive tape, with mannitol 20% (Samen Serum institute, Mashahd, iran) used in the intervention group and Ringer’s lactate serum in the control group. The colleague responsible for preparing and concealing the study serum had only one role in the study. The prime solution in the intervention group consisted of 200 ml of mannitol 20%, 900 ml of Ringer’s lactate serum, 50 ml of sodium bicarbonate, and 5000 units of sodium heparin. In the control group, the prime solution included 1100 ml of Ringer’s lactate serum, 50 ml of sodium bicarbonate, and 5000 units of sodium heparin. Additional Ringer’s lactate serum was added to the CPB circuit if needed during the procedure. Cardiac protection was achieved through tepid St Thomas cardioplegia for all patients, with non-pulsatile blood flow (2.4 mL/m2) used. Patients underwent moderate hypothermia (28-32 C), and blood pressure was maintained at 50-80 mm Hg during the procedure. TNG serum or phenylephrine was used as needed to regulate blood pressure.

 If a patient’s hematocrit dropped below 24%, packed red blood cells were administered to increase it to above 24%. For patients with a low hematocrit at the start of the operation, compressed red blood cells were added to the system before CPB began. This ensured that after diluting the blood in the system, the hematocrit would be above 24%. Once the patient’s hemodynamics were stable post-operation, they were separated from the CPB. Throughout the study, colleagues from various teams were kept blind to the patient group, and the surgeon remained the same individual.

###  Data collection

 Before the operation, we recorded the patient’s age, sex, body surface area (BSA), history of blood pressure, diabetes, smoking, serum creatinine, and blood urea nitrogen(BUN). The amount of diuresis was recorded during the operation, as well as on the first, second, and third day following the operation.

 Additionally, the aortic cross-clamp time and CPB time were recorded during the operation. Serum levels of creatinine and BUN were monitored 30 minutes, 24 hours, and 48 hours after the operation using an arterial sample. We also recorded the duration of mechanical ventilation, length of stay in the intensive care unit, and 30-day in-hospital mortality.

###  Statistical analysis

 Data was analyzed using SPSS software (version 22). To analyze the results of this study, the first step was to check the distribution of the data in terms of normality using the Kolmogorov-Smirnov test. Differences between independent groups were analyzed using the Student’s t-test for normally distributed variables and the Mann-Whitney U-test for variables that were not normally distributed. Categorical data were presented as percentages or numbers of observations, while continuous variables with a normal distribution were presented as means ± standard deviation (SD). Chi-square was used to check the difference between categorical variables. Repeated measures analysis of variance was utilized to examine how the main variables changed over four time periods. The Greenhouse-Geisser correction was adjusted for lack of sphericity in repeated measures. Data analysis was conducted by an individual who was blinded to the groups. A *P* value of < 0.05 was considered significant

## Results

**Figure 1:**
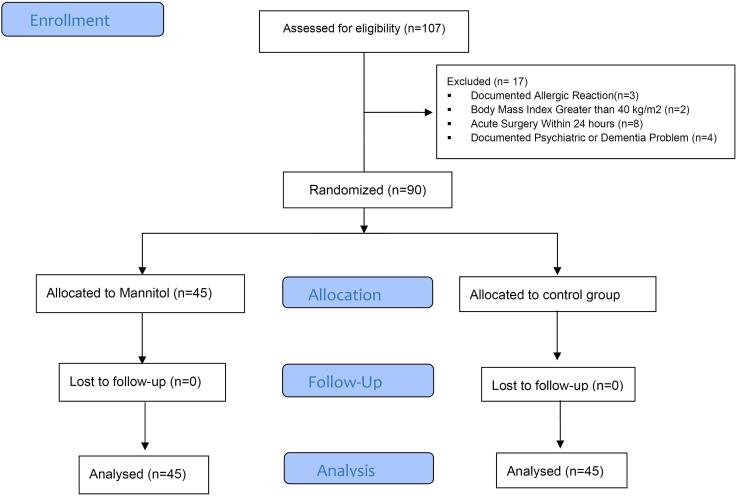
Patients flowchart

 During this study, a thorough patient selection process was implemented. Out of the initial 107 eligible patients, three were excluded due to documented allergic reactions, two due to a Body Mass Index greater than 40 kg/m^2^, eight due to acute surgery within 24 hours, and four due to documented psychiatric or dementia problems. This meticulous approach ensured that the study proceeded with a robust sample size of 90 patients (45 patients in the intervention group and 45 patients in the control group), as illustrated in Figure 1. Notably, no participant was excluded from the study after the intervention began, further enhancing the reliability of the findings.

 The basic information of the participants is presented in Table 1. The table indicates that the demographic characteristics and risk factors between the control and intervention groups were not statistically significant (P > 0.05, which means the difference observed could be due to random chance), except for blood pressure, which was identified as a confounding factor in the data analysis and was adjusted (*P* = 0.008, which indicates a significant difference). Therefore, the groups studied were homogeneous regarding the baseline characteristics. Table 1 also reveals no statistically significant difference between the intervention and control groups regarding CPB time, aortic cross-clamp time, duration of mechanical ventilation, 30-day mortality, and length of stay in the ICU (*P* > 0.05, indicating no significant difference).

**Table 1:** Comparisons of baseline characteristics between the two groups

**Variables**		**Control group (n=45)**	**Intervention group(n=45)**	***P*** ** Value**
Sex	Male	30(66.7)	28(62.2)	0.41*
	Female	15(33.3)	17(37.8)	
Age (years)		65.6 ± 9.08	62.68 ± 8.05	0.11**
Smoking		15(33.3)	17(37.8)	0.41^*^
Diabetes		20(44.4)	17(37.8)	0.33^*^
Hypertension		41(91.1)	31(68.9)	0.008^*^
CPB time (min)		89.95 ± 16.09	90.04 ± 17.36	0.98^**^
BSA(m^2^)		1.79 ± 0.16	1.82 ± 0.21	0.53^**^
Cross clamp time (min)		55.91 ± 10.92	56.31 ± 11.45	0.86^**^
Ventilation time (hour)		6.77 ± 1.49	6.57 ± 1.49	0.52**
30-d mortality		2(4.4%)	1(2.22%)	0.50†
ICU length of stay (hour)		18.46 ± 1.28	17.68 ± 2.81	0.90†

Abbreviations: BSA. Body Surface Area; CPB, Cardiopulmonary Bypass; ICU, intensive care unit * Chi-square test ** Independent t-test † Mann–Whitney U Values are mean ± SD or n (%)

 Based on the results of variables indicating renal function shown in Table 2, such as diuresis, creatinine, and BUN, there was no significant difference in serum creatinine between the two groups at any time (*P* = 0.53). Similarly, there was no significant difference in BUN levels between the two groups at any time (*P* = 0.13). According to the study results, there was no statistically significant difference in the trend of diuresis changes between the two groups (*P* = 0.10). These findings underscore the importance of our research, as they have direct implications for patient care and treatment decisions.

**Table 2:** Changes in Serum Cr, Serum Bun, and Diuresis

**Variables**		**Control group (n=45)** **mean±SD**	**Intervention group(n=45)** **mean±SD**	***P *** **value**
SCr	Baseline	1.12 ± 0.30	1.09 ± 0.21	0.53^*^
	30 minute postprocedure	1.12 ± 0.30	1.04 ± 0.27	
	24 hours postprocedure	1.34 ± 0.36	1.23 ± 0.35	
	48 hours postprocedure	1.34 ± 0.41	1.23 ± 0.41	
SBun	Baseline	20.06 ± 8.22	18.78 ± 5.96	0.13^*^
	30 minute postprocedure	19.66 ± 5.97	17.55 ± 6.45	
	24 hours postprocedure	24.37 ± 8.77	23.26 ± 7.99	
	48 hours postprocedure	27.46 ± 8.93	29.06 ± 12.52	
Diuresis	during operation	2304 ± 1093.43	2850 ± 1007.92	0.10^*^
	Day 1	3178.44 ± 1059.99	3256 ± 1183.06	
	Day 2	3712.22 ± 912.36	3145.33 ± 820.56	
	Day 3	3712.22 ± 1047.73	3035.55 ± 1571.82	

Abbreviations: SCr. serum creatinine; SBun. Serum blood urea nitrogen * Repeated measures analysis of variance Values are mean ± SD

## Discussion

 This study demonstrated that using mannitol in Prime solution does not impact on postoperative diuresis, renal function, or mortality, however it was found that it increases intraoperative diuresis. Many patients undergoing elective CABG, are at risk of postoperative renal dysfunction and renal failure, which can lead to prolonged hospital stays and increased mortality rates in intensive care unit.^20^ Although the likelihood of developing postoperative renal dysfunction in patients without prior renal issues is relatively low, the results show that CABG significantly increased mortality, complications, and length of stay. Even a slight increase in preoperative serum creatinine level (> 1.2 mg/dL) can substantially raise mortality and complications.^21^ It is also stated that increased serum creatinine and BUN, as well as the presence of diabetes and obesity are considered as strong indicators of acute renal injury in cardiac surgery.^22^ Despite systemic oxygen is delivered during CPB, the renal oxygen supply/demand relationship is disturbed, and renal oxygenation worsened even after completion of CPB.^23^

 CPB leads to renal vasoconstriction and bleeding, which can impair renal oxygenation. This, along with the increased release of tubular damage markers, can further exacerbate the damage to the kidneys during and after cardiopulmonary bypass.^23^

 Mannitol can redistribute blood flow to the kidneys by causing vasodilation of the renal vessels. However, this action does not affect the amount of renal oxygenation or filtration fraction. Instead, it increases the balance of perfusion-filtration and supply-demand of oxygen. ^24^ In the study by Moreira et al it was found that there is a significant positive relationship between the occurrence of acute kidney failure in patients after CPB, the age of the patients, and the administration of mannitol and furosemide during the operation.^25^ Among the complications of mannitol injection, acute kidney failure can be mentioned;^26^ the incidence rate of this complication has been reported as 13.6%.^27^ In the present study, no diuretic was used during the operation except for mannitol. The mean intraoperative diuresis in patients receiving mannitol was higher than in the control group. However, the mean diuresis after the operation did not show a significant difference between the two groups. This could indicate the short-term effects of mannitol on the kidneys.

 The present study showed that the use of mannitol in Prime solution has no impact on the level of BUN and creatinine despite the increase in intraoeperative diuresis. Ljunggren et al have also stated in their study that after the use of mannitol in CPB prime, they did not observe any significant difference between the two groups in terms of renal parameters and fluid balance,^20^ which is in line with the findings of the present study. Mannitol has little effect on renal function in patients with normal postoperative creatinine.^28^ Also, the findings of previous studies indicate that the use of mannitol after partial nephrectomy has no benefit,^29^ and its administration has no short-term or long-term renal benefit.^30^

 Using hyperosmolar solutions in prime of CPB can cause adverse effects in organs, such as osmotic demyelination syndrome, by changing plasma osmolality.^6^ Also, due to the inhibition of sodium absorption in renal tubules, mannitol can cause a decrease in sodium level^15^ and cause hyponatremia complications. The above evidence limits the use of mannitol in CPB. In the present study, no statistically significant difference was observed in 30-day mortality between the two groups, which is in the same direction as Hamiko et al’s study.^11^ In a study conducted by Haydock et al, the mortality rate between patients receiving mannitol and those not receiving mannitol did not have a statistically significant difference.^17^ This finding was also stated in the study by Binder et al,^31^ which agrees with the current study’s results.

 In this study, there was no statistically significant difference between the two groups regarding the length of stay in the intensive care unit and the duration of connected to mechanical ventilation. This finding aligns with study conducted by Haydock et al who concluded that removing mannitol from the Prime solution does not have a statistically significant effect on the length of stay in the intensive care unit, hospital, or the duration of mechanical ventilation.^17^ Meanwhile, Hamiko et al stated that mannitol infusion is associated with a shorter duration of mechanical ventilation, intensive care unit, and hospital stay.^31^ In a study conducted by Shim et al the average duration of hospitalization in the intensive care unit and the duration of mechanical ventilation were significantly lower in the group receiving mannitol.^32^ In 2019, the results of Ljunggren et al’s study contradicted the findings of other studies by stating that patients receiving mannitol significantly had a longer average length of stay in the intensive care unit.^33^ There were various confounding factors that could have significantly impacted on the final results; However, via blinding process and randomization of participants, we were able to decrease their effects on the results; The second limitation of this study was that it was single-centered. Another limitation was that the possibility of a type II error could not be ruled out.

## Conclusion

 This double-blind, randomized controlled trial study showed that adding serum mannitol to the priming solution of patients undergoing coronary artery bypass surgery does not affect renal function, mechanical ventilation duration, length of ICU stay, and 30-day mortality. Therefore, it is concluded that serum mannitol cannot be used as a preventive measure for patients with acute kidney failure following heart surgery.

## Acknowledgements

 This study was conducted as part of a master’s thesis, which Urmia University of Medical Sciences granted. The authors would like to express their gratitude to the cardiac surgery team at Shahid Madani and the cardiovascular, medical, and research centers. We would like to give special thanks to Dr. Reza Nematollahi Maleki for the effort he put on this work regarding editing.

## Competing Interests

 The author(s) declared no potential conflicts of interest concerning this article’s research, authorship, and publication.

## Ethical Approval

 The ethics committee of Urmia University of Medical Sciences approved the current study (IR.UMSU.REC.1401.319).
